# Heat and flow dynamics in cities: an experimental comparative study across diverse urban morphologies

**DOI:** 10.1098/rsta.2024.0573

**Published:** 2025-11-06

**Authors:** Yunpeng Xue, Yongling Zhao, KaMing Wai, Chao Yuan, Jan Carmeliet

**Affiliations:** ^1^ Singapore-ETH Centre, ETH Zurich, Singapore; ^2^ Department of Mechanical and Aerospace Engineering, Monash University, Melbourne, Australia; ^3^ Department of Mechanical and Process Engineering, ETH Zurich, Zürich, Switzerland; ^4^ Department of Architecture, National University of Singapore, Singapore; ^5^ NUS Cities, National University of Singapore, Singapore

**Keywords:** urban flow, urban morphology, buoyancy-driven flow, PIV/LIF

## Abstract

Urban areas are renowned for their intricate atmospheric dynamics, influenced by diverse building configurations. Understanding the implications of urban morphology for flow patterns, ventilation and heat dissipation is crucial for urban climate management. However, comprehending the interplay between thermal-driven buoyancy flow and urban morphology remains a challenge. To address this gap, we measured heat transport and fluid flow around three-dimensional parametric urban models resembling Singapore's urban morphology accounting for buoyancy effects, using simultaneous Particle Image Velocimetry (PIV) and Laser-Induced Fluorescence (LIF) measurements. Our study meticulously documents the development of non-isothermal urban flow, highlighting heat plume generation from the ground, buoyant updraft development and temperature variations along the flow. The variations in urban morphologies have a profound impact on these developmental processes, resulting in substantial differences in heat and flow mechanisms, ventilation efficiency and heat removal performance. For example, significant differences are observed in ventilation rates and their fluctuations, with values reaching up to approximately 10.5 times and 12.2 times, respectively. These findings of the fluid flow and heat spreading above the ground contribute to the broader understanding of urban heat dynamics by demonstrating how localized thermal effects propagate through urban environments, influencing microclimatic conditions.

This article is part of the theme issue ‘Urban heat spreading above and below ground’.

## 1. Introduction

The relentless march of urbanization has cast a glaring spotlight on the environmental and climatic conditions within cities, where the heat island effect looms large [1,2]. Unlike natural meteorological phenomena such as heat waves, wind patterns and humidity, the configuration of urban spaces is a factor which can be humanly controlled. Urban morphology, the layout and structure of cities, wields profound sway over local climate dynamics, affecting thermal comfort, pollutant dispersion, public health, energy consumption and the overall sustainability of urban areas [3,4]. Consequently, urban planning and design must meticulously consider urban morphology to mitigate adverse environmental impacts, particularly by examining the spread of heat above the ground and its isolated and coupled effects across scales to fully understand the impact of urban heat islands.

A nuanced understanding of heat and fluid flow characteristics within urban areas is indispensable for effective urban development and design [5]. Researchers have employed a variety of methodologies to investigate these phenomena and mechanisms, ranging from field measurements in diverse urban settings to laboratory-scale fluid tunnel experiments and numerical simulations of selected urban regions.

Field measurements, capturing parameters such as wind velocity, humidity, air and land surface temperatures, and pollutant levels, provide invaluable insights into the urban climates of real cities. For instance, Li *et al.* [6] conducted a comprehensive study integrating morphological parameters from an aerodynamic perspective to discern local ventilation efficiency within heterogeneous urban areas. Their analysis of weather station data across different urban morphologies in Wuhan, China, revealed distinct airflow behaviours and delineated local ventilation performance zones. It has also been reported that both two-dimensional (planar) and three-dimensional (including variable height) urban morphology significantly influence thermal environments, with two-dimensional metrics, particularly composition metrics, showing stronger correlations with land surface temperature (LST) [7], while three-dimensional morphology, which incorporates height as a key parameter, plays a more critical role in predicting air temperature, especially in high-rise dominated areas [8].

Due to the inherent limitations of field measurements, such as uncontrollable parameters and fixed urban morphology in each test, laboratory-scale experimental studies serve as crucial tools in advancing our comprehension of the intricate multi-physical processes within urban street canyons. By replicating controlled scenarios in a laboratory environment, typically within a fluid tunnel, researchers can isolate and measure specific variables and phenomena, thereby offering valuable insights into the underlying mechanisms. A meticulous wind tunnel experiment employed a realistic 1:200 scale morphological model of Nantes, France, featuring a diverse array of upstream buildings [9]. This study reaffirmed the presence of the amplitude modulation mechanism observed in previous analyses of both smooth and homogeneous rough wall boundary layers. Notably, this mechanism persisted even in the more intricate morphological model featuring a single upstream building of relatively low height, albeit with some modifications.

Another study investigated pollutant dispersion around a realistic cluster of tall buildings, representing a Beijing neighbourhood [10], employing Particle Image Velocimetry (PIV) and Planar Laser-Induced Fluorescence (PLIF) based flume measurements at two scales. The research revealed that tall buildings amplify the depth of the urban canopy layer and introduce pronounced flow disturbances in the roughness sublayer, overriding disparities in initial flow conditions and the influence of low-rise structures. Below the mean canopy height, significant advective fluxes in the lateral direction pose challenges for modelling due to their geometric complexity.

Numerical simulation offers flexibility in controlling variables such as wind velocity, direction, temperature and urban morphology. With careful grid resolution selection, simulations can capture dominant flow structures in different configurations [11,12]. For instance, Javanroodi *et al.* [13] utilized a hybrid model coupling Computational Fluid Dynamics (CFD) with Artificial Neural Networks (ANN) to predict interactions between climate variables and urban morphology. This approach quantified the vertical structure of mean wind speed and air temperature across idealized urban areas of Stockholm, Sweden, thereby enhancing urban design and energy performance amidst climate change. Additionally, the correlation between thermal conditions and urban spatial configuration are explored using ENVI-met [14], highlighting a greater daytime influence of urban morphology on local thermal conditions, varying correlations with different statistical approaches.

Meanwhile, turbulent flow and dispersion characteristics in a complex urban street canyon in Seoul, Republic of Korea, were investigated using Large Eddy Simulation (LES) [15]. The study revealed active turbulence near building tops during parallel wind flows, and heightened turbulence within the street canyon during oblique or perpendicular winds. These findings align with previous studies [9,10,16], emphasizing the influence of high-rise buildings on surrounding airflow and dispersion patterns, underscoring the importance of detailed airflow and dispersion modelling in urban development involving tall structures.

Due to the intricate nature of urban geometries, fully representing all details of a realistic city remains a daunting task. Therefore, idealized geometries are often utilized for parametric investigations, particularly when thermal conditions become significant, as buoyant flow induced by surface heating can alter flow behaviour, heat transfer and ultimately impact thermal comfort. Chen *et al.* [17] conducted numerical simulations to analyse the outdoor ventilation efficiency and airflow structures of specified street canyons within an idealized building array with surface heating. Their results revealed significant differences in airflow structures within the three-dimensional building array compared to previous two-dimensional models and isolated street canyon models, particularly under oblique wind conditions. These significant differences of flow behaviour and thermal performance of the two- and three-dimensional street canyons in non-isothermal condition is also emphasized in a recent experimental study [18]. Wai *et al.* [19] coupled a Computational Fluid Dynamics (CFD) model with a street-level outdoor thermal comfort model to explore the relationship between airflows, air temperature, building morphologies and outdoor human thermal comfort. The study suggested reducing sensible street-level physiologically equivalent temperatures through better building design, emphasizing the effectiveness of reducing building lengths and increasing low-level porosity in mitigating heat stresses at the pedestrian level.

Recently, Allegrini *et al.* [20–22] simulated the local microclimate using surface temperatures determined by the Building Energy Simulation (BES) model as boundary conditions. Their findings unveiled a complex interaction between buildings and the local microclimate, with urban morphologies featuring cubical or elongated buildings exhibiting similar surface temperatures across different façades. Buoyancy was found to be crucial for low wind speed scenarios, with upstream buildings exerting a strong influence on downstream heat fluxes and temperatures. Furthermore, investigations conducted in a wind tunnel [23] highlighted the importance of considering full three-dimensional flow structures in street canyons, with building configurations significantly impacting wind flow patterns and heat removal. Studies employing Particle Image Velocimetry (PIV) and Laser-Induced Fluorescence (LIF) techniques [24,25] provided accurate measurements of velocity and temperature profiles in simplified street canyons with ground heating, shedding light on heat and fluid flow mechanisms and the impacts of building morphology. These findings underscore the diverse flow behaviours, ventilation rates and heat removal performances resulting from variations in street canyon configurations.

The significant influence of urban morphology on local climate, including ventilation, heat removal, pollutant dispersion and thermal comfort, has garnered considerable research interest due to its importance in urban design, heat island mitigation and public health improvement. However, understanding the impacts of urban morphology, particularly those involving thermal-driven buoyancy flow, remains a challenge. Despite ongoing research efforts, a comprehensive understanding of the interplay between heat transport and fluid flow in complex urban environments remains elusive. In this study, we aim to bridge this knowledge gap by experimentally investigating how different urban morphologies influence the combined effects of ground heating and buoyancy-driven flows, thereby advancing our understanding of urban heat spreading dynamics. Using three-dimensional parametric urban models that closely resemble Singapore’s actual urban morphology while accounting for buoyancy effects, we conducted high-resolution heat and fluid flow measurements. Through simultaneous Particle Image Velocimetry (PIV) and Laser-Induced Fluorescence (LIF) measurements in a large closed-circuit water tunnel, we captured detailed heat transfer and flow dynamics across various configurations. Our analysis of heat and flow behaviours in different urban morphologies allowed us to identify the impacts of canyon configuration, which are detailed in the following sections.

## 2. Experimental configurations

### (a) Experimental facilities

This study involves simultaneous Particle Image Velocimetry (PIV) and Laser-Induced Fluorescence (LIF) measurements of thermal flow among different urban morphologies in the ETH Zurich Atmospheric Boundary Layer Water Tunnel. The water tunnel is equipped with a 110-kW pump capable of producing water speeds ranging from 0.02 to 1.5 m s^−1^. It features a development section that measures 6 m long and a measurement section with a cross-sectional area of 0.6 × 1 m².

PIV measurements are obtained by employing a Litron 100 Hz Nd-YAG laser (532 nm) to excite the dye and illuminate the field-of-view. 10 micron hollow glass is used to seed the water flow with a particle density of approximately 40 to 60 in an integration window. To obtain temperature-dependent fluorescence, uranine is utilized due to its minor pulse-to-pulse intensity variation when exposed to the laser. The setup includes two sCMOS 16-bit dual-frame cameras with a resolution of 2048 × 2048 pixels² at 15 Hz. These cameras are aligned in the spanwise direction of the tunnel to focus on the same measurement plane. As the emission peak of uranine occurs at a wavelength of 510 nm when excited at 532 nm, a bandpass filter of 535−630 nm is adopted to capture the fluorescence.

**Figure 1.**
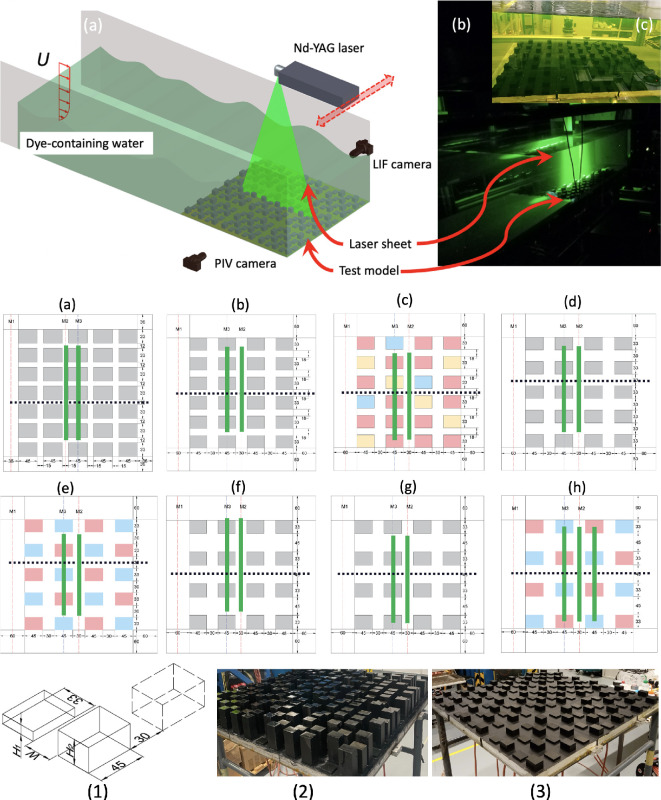
Top: (a) water tunnel filled with dye-containing water and equipped with a PIV-LIF measurement system. (b and c) Actual measurement setup showing the laser sheet and building models submerged in the water tunnel. Middle: Illustration of the different urban morphologies tested in the current study, with detailed information provided in table 1. The green solid lines denote the locations of the laser sheets, while the black dashed lines represent the focused street canyon. Bottom: A detailed illustration of the model dimension of case e (1) and actual tested urban model of case c (2) and case h (3).

To replicate urban thermal conditions, nine stainless-steel plates, each measuring 330 mm × 330 mm, are employed, with conductive building models placed on the surfaces. Beneath each plate, electrical heating elements are installed within sealed cavities to prevent direct contact with water. Thermocouples are incorporated into each plate to provide real-time temperature data. The experimental setup in the water tunnel, including the laser, cameras and building models, is presented in figure 1.

### (b) Parametric model configuration

To establish the parametric models, which are representative of Singapore scenarios with various urban densities, we calculated urban morphology indices, i.e. site cover ratio ($\lambda_{p}$), average height (*H*) and sky view factor ($\Psi_{sky}$) in a GIS platform with a resolution of 600 m × 600 m. Satellite data of Singapore’s building geometry, proven to be accurate and effective [26,27], is utilized to calculate these parameters. The site coverage ratio is determined by dividing the building footprint area by the site area, while the average height represents the mean value of building heights (weighted by building footprint area). The sky view factor is computed within a 100 m radius of the sky hemisphere using a raster-based algorithm with a resolution of 1 m × 1 m, aggregated into 600 m × 600 m. The selected urban morphologies comprise nine different configurations, spanning from low-density urban to high-density urban, with detailed information provided in table 1. All nine heat plates feature identical building model arrays, with measurements conducted on the central plate. The green solid lines represent the field-of-view (FOV) locations for the PIV-LIF measurements in each scenario, while the black dashed lines denote the focused street canyons for quantitative analysis in subsequent sections. The M3 and M2 planes represent the centrelines of the canyon and street, respectively. All building models possess the same cross-section but differ in height. Grey building models indicate uniform height across all models in the test, while pink and blue denote high and low building models in each configuration, respectively (see detailed information in table 1). In case (c), yellow marks indicate the lowest model with a height of 9 mm. A detailed illustration of the canyon configuration, along with examples of two selected urban models studied in this paper (cases c and h), is also shown in figure 1.

**Table 1.** Geometrical parameters of the tested urban models. *H*, *H*_1_ and *H*_2_ are the heights of the building models, *W* denotes the spacing between the two building models, i.e. the width of the canyon.

case	description	*λ*_*p*, Real_	*λ*_*p*, Model_	*H*_Real_	*H*_Model_	*Ψ*_Real_	*Ψ*_Model_	height	Canyon width	*H*/*W*	Ri
a	high-density (large building footprint)	0.46	0.46	9.4	9	0.33	0.36	*H* = 9 mm	*W* = 12 mm	0.75	0.42
b	high-density (high-rise buildings, uniform building height)	0.32	0.3	84.1	84	0.41	0.4	*H* = 84 mm	*W* = 18 mm	4.7	3.92
c	high-density (high-rise buildings, building height variance)	0.32	0.3	84.1	84	0.41	0.42	*H*_1_ = 9 mm *H*_2_ = 84 mm *H*_3_ = 99 mm	*W* = 18 mm	4.7	3.92
d	medium density (uniform building height)	0.27	0.29	24.4	24	0.5	0.5	*H* = 24 mm	*W* = 30 mm	0.8	1.12
e	medium density (building height variance)	0.27	0.29	24.4	24	0.5	0.51	*H*_1_ = 36 mm *H*_2_ = 12 mm	*W* = 30 mm	0.8	1.12
f	low density (uniform building height)	0.21	0.23	17.7	24	0.6	0.56	*H* = 24 mm	*W* = 45 mm	0.53	1.12
g	low density (uniform building height)	0.21	0.23	17.7	18	0.6	0.59	*H* = 18 m	*W* = 45 mm	0.4	0.84
h	low density (building height variance)	0.21	0.23	17.7	18	0.6	0.6	*H*_1_ = 27 m *H*_2_ = 9 m	*W* = 45 mm	0.4	0.84

### (c) Measurement procedures

The water velocity in the tunnel for this study is set at approximately 0.03 m/s, with minor variations (<2%) due to blockage of the test model and water evaporation. Velocity fields are acquired from Particle Image Velocimetry (PIV) measurements using a Dantec system, processed through cross-correlation with an integration window of 32 × 32 pixels² (equivalent to 10 pixels/1 mm). Temperature profiles are derived from post-processing Laser-Induced Fluorescence (LIF) images. As the local intensity of the LIF image is influenced by uranine concentration, laser intensity and fluid temperature, maintaining stable laser power and constant uranine concentration ensures a linear relationship between measured local intensity and fluid temperature in a uniformly mixed uranine flow. Calibration of this relationship is performed daily to ensure measurement reliability, considering the decrease in dye’s excitability over time. Additionally, several thermocouples measure ground and fluid temperatures, aiding in validation and correction of temperature measurements.

Simultaneous measurement of velocity and temperature is achieved by overlaying the two fields from the same Field of View (FOV). For each test configuration, 1500 pairs of images are captured at a frequency of 15 Hz, resulting in a recording time (*T*) of 100 s for statistical analysis. Velocity field uncertainty is estimated to be 10^−5^ m/s using integration window-based statistics with sub-pixel accuracy at 1/10 pixel, which is two orders of magnitude smaller than the freestream velocity. Temperature measurement uncertainty depends on uranine emission spectrum, optical setup, and pulse-to-pulse laser intensity variation. The intensity-to-temperature ratio is approximately 450, resulting in an instantaneous temperature field uncertainty of 0.002°C. For time-averaged statistics, pulse-to-pulse laser intensity variation (2.02%) yields an uncertainty of around 0.09°C in the current study under heating conditions at 41°C. Further details of the measurement process are available in [25].

When describing non-isothermal flow, the bulk Richardson number is used to express the ratio of the buoyancy term to the flow shear term as [25]:


(2.1) $$Ri=\frac{g\beta ∆TH}{U_{f}^{2}}$$

where ∆*T* is the temperature difference between the floor and the freestream water, *β* denotes the thermal expansion coefficient of water, *g* is the acceleration due to gravity, *H* represents the height of the downstream building model and *U_f_* is the freestream velocity. The selection of our experimental parameters is guided by the scaling of the Richardson number. Taking these considerations into account, we opted for a representative real urban climate scenario featuring a wind speed of 2.7 m/s and a temperature difference of 10°C between the surface and the ambient air.

## 3. The flow mechanism in different morphological urban areas

### (a) Understanding the developing buoyant flow

**Figure 2.**
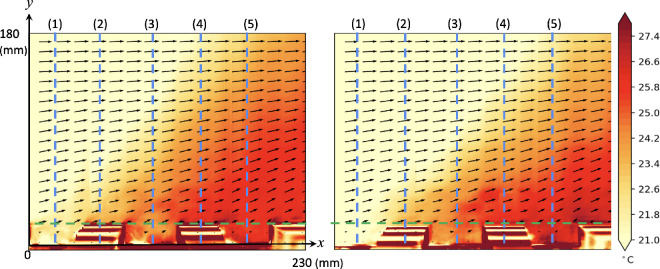
Averaged temperature and velocity vector fields along the centreline of the canyon (left) and street (right) with a freestream velocity of 0.03 m/s and a floor temperature of approximately 41°. Vertical blue dashed lines and a horizontal green dashed line at the canyon roof level indicate the locations for further quantitative presentation of the results in figures 3–5.

We begin by examining the flow and temperature patterns along the central plane of the canyon and the street, as depicted in figure 1 (M3 and M2). For example, figure 2 illustrates the average temperature and velocity vector fields, with a field of view measuring 230 mm in width and 180 mm in height, along the centreline of the canyon (left) and street (right) of case (g) (*H* = 18 mm, *W* = 45 mm), with *U*_*f*_ = 0.03 m/s and Ri ≈ 0.84. Distinct heated buoyant flows are observed, generated from the heated ground and building surfaces, resulting in a significant increase of 5~6 °C in water temperature near the ground surface. Some of the accumulated heat is carried away by the updrafts and subsequently flushed downstream by the approaching flow above the rooftops. While most of the flow and temperature fields along the canyon and street centrelines exhibit similarities, notable differences exist between the flow within the canyon region and that within the street. Detailed flow behaviour within the street canyons with different configurations has been reported elsewhere [24]. Further quantitative analysis of heat and fluid flow along the canyon and the street is presented in the subsequent sections.

**Figure 3.**
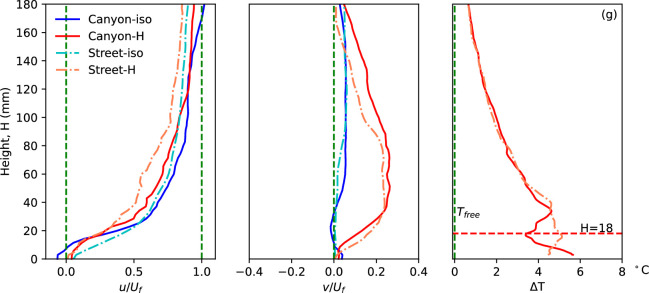
The streamwise and vertical velocities, normalized by the freestream velocity, along with the temperature rise along the centreline (location 3) of the street canyon and a parallel position along the street under both isothermal (-iso) and heating conditions (-H) in case (*g*) are presented.

**Figure 4.**
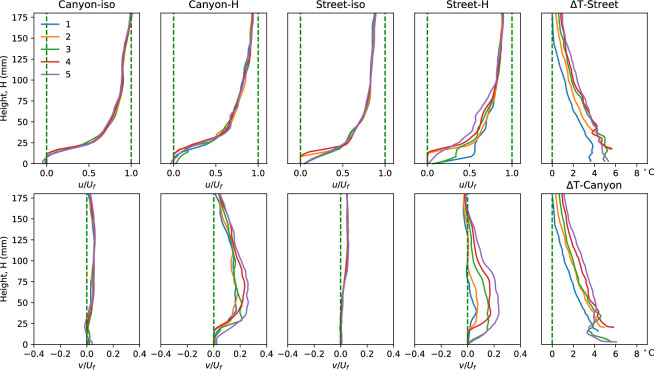
The flow properties at different locations of the tested urban model (locations 1–5 as shown in figure 2) illustrate the gradual thermal impacts on the horizontal velocity, vertical velocity and fluid temperature.

**Figure 5.**
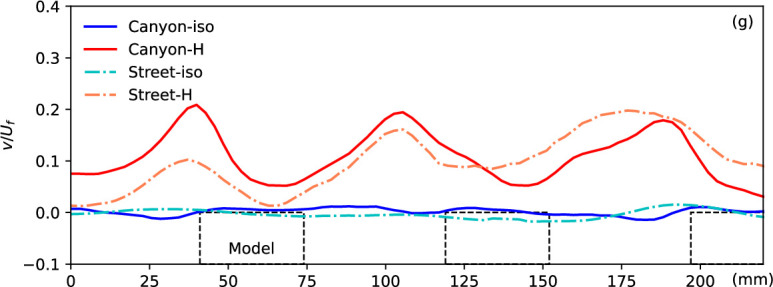
The normalized vertical velocity at the canyon roof level along the flow in different scenarios, as indicated by the green dashed line in figure 2. The locations of the models are presented by the dashed blocks.

The streamwise and vertical velocity components along the centreline (line 3 in figure 2) as functions of height in all different scenarios are normalized by the freestream velocity and summarized in figure 3. Additionally, the temperature rise in the heating condition along the vertical canyon and street centrelines is plotted in figure 3, demonstrating the impacts of heating and canyon configuration on heat flux performance. The streamwise velocity exhibits slightly different distributions in the four conditions. ‘Canyon-iso’ represents the velocity profiles along the canyon centreline in the isothermal condition, while ‘Street-H’ indicates the street central plane with the ground floor heated to 41°C. In the isothermal condition, flow near the ground is accelerated along the street, as evidenced by significantly higher velocity compared to canyon flow, a phenomenon commonly observed in real-life scenarios. The only negative region of streamwise velocity is observed near the ground, i.e. inside the canyon, in the isothermal flow condition, representing the existence of a canyon vortex. Flow over the canyon is slightly faster than over the street in both isothermal and non-isothermal conditions, as shown by the streamwise velocity in figure 3. This is primarily due to the blockage effect of the building models, which reduces the local cross-sectional area available for flow and consequently accelerates the velocity over the canyon to maintain a constant volumetric flow rate within the confined tunnel. When the ground is heated, flow over both the canyon and street decelerates due to the constant momentum from the mixing of the updraft buoyant flow, evident in the significant positive vertical velocity (*v/U_f_*) in the heating condition. The buoyant flow originates from the heated ground and becomes significant over the street canyon, indicating the substantial impact of high-temperature ground on local flow mechanism and heat flux at higher altitudes.

The temperature rise of the flow in the non-isothermal condition exhibits a different distribution near the floor, i.e. in (*H* < 18 mm) and over the canyon (18 mm < *H* < 40 mm), compared to the reference temperature profile in the freestream flow [18,28]. The temperature profile along the canyon plane, particularly the zigzag pattern in the lower region, is determined by the complex flow structure within the canyon. Meanwhile, the temperature along the street shows a different pattern close to the ground. These differences, caused by urban configuration, underscore the impacts of urban morphology on flow temperature.

Figure 4 summarizes the flow properties (normalized horizontal and vertical velocity components and temperature rise) at different streamwise locations to illustrate the flow development process. Locations 2 and 4 are distinguished from the rest as they represent positions on the building models, thus only providing effective data from the building top. On the other hand, locations 1, 3 and 5 depict flow results close to the ground in the canyon.

The first four subplots in the top row display the normalized streamwise velocity in different scenarios, with the notable difference observed in the flow along the street, particularly within the canyon area. This difference becomes more significant when the floor is heated, extending over a larger region from the ground, with the thickening boundary layer clearly visible along the street. The flow development can also be observed from the fluid temperature rise (Δ*T*) along both the canyon and street centrelines in the streamwise direction, as depicted in figure 2, with the quantitative results plotted here indicating a gradual increase in fluid temperature. The buoyant updraft, evident through the vertical velocity in the canyon and the street, signifies the significant force induced by the higher ground temperature, impacting up to higher levels from the floor. As the flow passes through the high-temperature urban area, it gradually experiences the effects of buoyant flow, transferring heat from the surface to the cooler flow at higher levels. The disturbance caused by the building models and canyons illuminates this developing influence more clearly, particularly evident in the vertical velocity along the street, which also indicates the growing region influenced by the thermal factor along the flow.

Figure 5 presents the vertical velocity component along the roof level of the canyon and street, as indicated by the green dashed line in figure 2. This vertical velocity at the canyon opening level provides crucial insights into the ventilation condition within the canyon or along the street, essential for understanding the mechanism. In the isothermal condition, minimal vertical flow is observed in the urban region, except in front of the building models where a slightly negative velocity indicates downward flow, contributing to the formation of the canyon vortex in such configurations. However, significant upwards flow is generated by the heated ground surface, with the maximum updraft observed near the windward side of the building models in the canyon. This updraft is influenced by both the surface temperature and canyon configuration. Interestingly, a similar vertical flow pattern is observed along the street, where strong updrafts occur at streamwise locations aligned with the windward canopy surfaces of the canyon. While these updrafts are not adjacent to the building façades themselves, their position suggests that, in addition to buoyancy-driven effects, the influence of upstream flow deflection and localized pressure buildup near the windward surfaces within the canyon may extend their impact laterally across the street. Moreover, the updraft gradually intensifies along the street, likely due to the downstream accumulation of thermal forcing. This vertical velocity trend is further explored in subsequent discussions under various canyon configurations.

**Figure 6.**
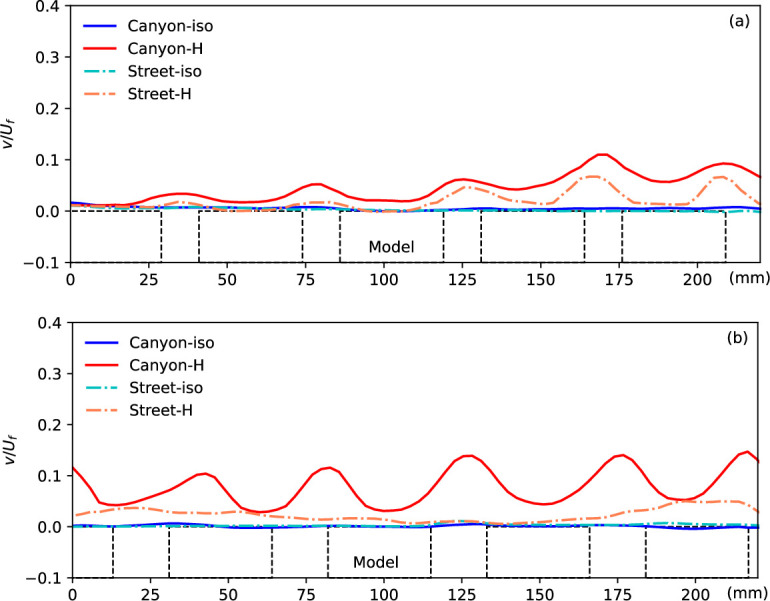
The normalized vertical velocity at the canyon roof level along the flow in different scenarios, as indicated by the green dashed line in figure 5. The locations of the models are presented by the dashed blocks.

It is noteworthy that the upwards flow along the street becomes more significant downstream, as indicated by the increasing vertical velocity and hence results in better ventilation and heat removal. This phenomenon is gradually enhanced by the accumulation of thermal force, as discussed in previous work, and explains that in urban areas, potential negative impacts can be induced by an upstream cooling source, e.g. an urban park or water body [25]. By contrast, for flow along the canyon, the accumulation is consistently impeded by the canyon, resulting in a repeatable pattern due to the repeated canyon configuration. This accumulation of thermal influence leads to more significant development of non-isothermal flow in narrow canyons or high aspect ratio canyons, as presented in figure 6 below.

### (b) Impacts of the canyon configuration

#### (i) Flow parameters within urban areas with different building height

**Figure 7.**
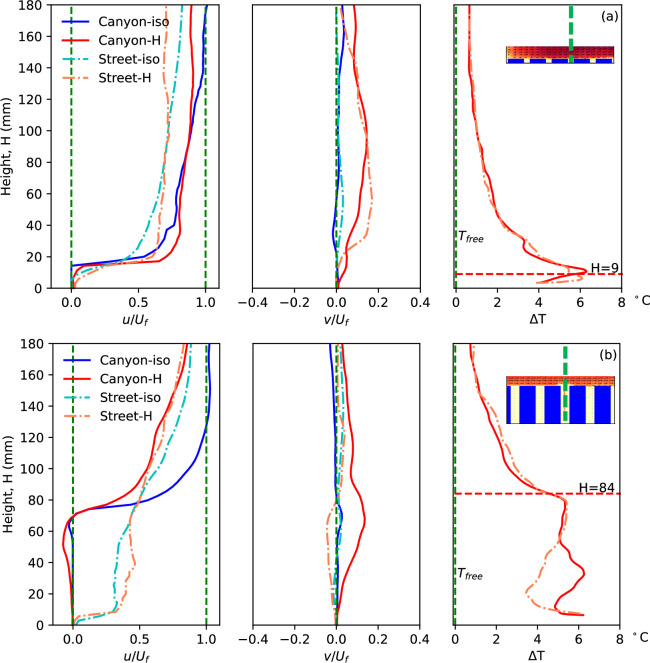
Normalized streamwise velocity, vertical velocity and the temperature rise along the centrelines in different urban morphologies, case (a) and (b). The blue dashed lines shown in the canyons indicate the locations of the plotted properties.

The urban morphology, particularly building configuration such as building height and canyon width, plays a pivotal role in shaping urban climate by determining urban flow characteristics, ventilation and heat removal performance. To assess the impacts of building height, a comparison between cases (a) and (b) is summarized in figure 7, covering flow velocity and temperature profiles along the vertical position, as well as vertical updraft buoyant flow along the horizontal direction.

In the high-density urban model with large building footprint, case (a) (*H* = 9 mm, *W* = 12 mm), similar streamwise velocity profiles are observed compared with the previously discussed case (g) (*H* = 18 mm, *W* = 45 mm), indicating slower flow along the street, particularly in the heating condition. This common characteristic is also evident in case (b) (*H* = 84 mm, *W* = 18 mm), both in the open area over the building models and street canyons, as well as in other tested scenarios. The most significant difference in horizontal flow is observed below the canyon roof level, where the interaction between canyon and street flows is strongly influenced by building height. In the case with low building models (a), the canyon flow is more directly affected by freestream penetration from above, resulting in a relatively small difference in streamwise velocity between the canyon and the adjacent street. The reduced velocity variation is further supported by the slower flow near the wall in the lower part of the boundary layer. By contrast, as building height increases (e.g. case b), the canyon becomes more isolated from the overlying flow, making it increasingly difficult for freestream air to reach into the canyon. This leads to a more pronounced velocity difference between the canyon interior and the street-level flow. Higher building models also result in weaker updraft of buoyant flow along the canyon, both in magnitude and region. Particularly along the street for case (b), downward flow is observed, indicating flow being drawn from the street near the ground into the street canyon, absorbing more heat and exhausting from the canyon opening.

In case (a), a notable deviation in the streamwise velocity profile is observed: the heated case exhibits a faster flow than the isothermal case up to approximately 80 mm in height. This trend is not seen in the other configurations. The distinctive urban morphology in this case—characterized by high building packing density, narrow streets, shallow canyons and uniformly low building heights—creates a surface that more closely resembles a rough wall than a typical urban canopy with well-defined street flow. Under isothermal conditions, this roughness induces a thicker boundary layer and leads to reduced streamwise velocity. However, when the surface is heated, strong buoyancy-induced updrafts appear to lift the incoming flow, thereby reducing its direct interaction with the rough elements below. This results in an effective decrease in aerodynamic roughness and enables a locally higher streamwise velocity near the surface. Similar mechanisms have been reported in the literature: uniform blowing over rough surfaces has been shown to reduce friction and pressure drag by forming a slip-like layer between the flow and surface [29]. Additionally, heating of rough surfaces has, in some cases, been found to increase near-wall velocity due to buoyancy effects [30]. These findings suggest that the observed behaviour in case (a) arises from the unique interplay between dense morphology and buoyancy-induced flow modification and merits further targeted investigation.

**Figure 8.**
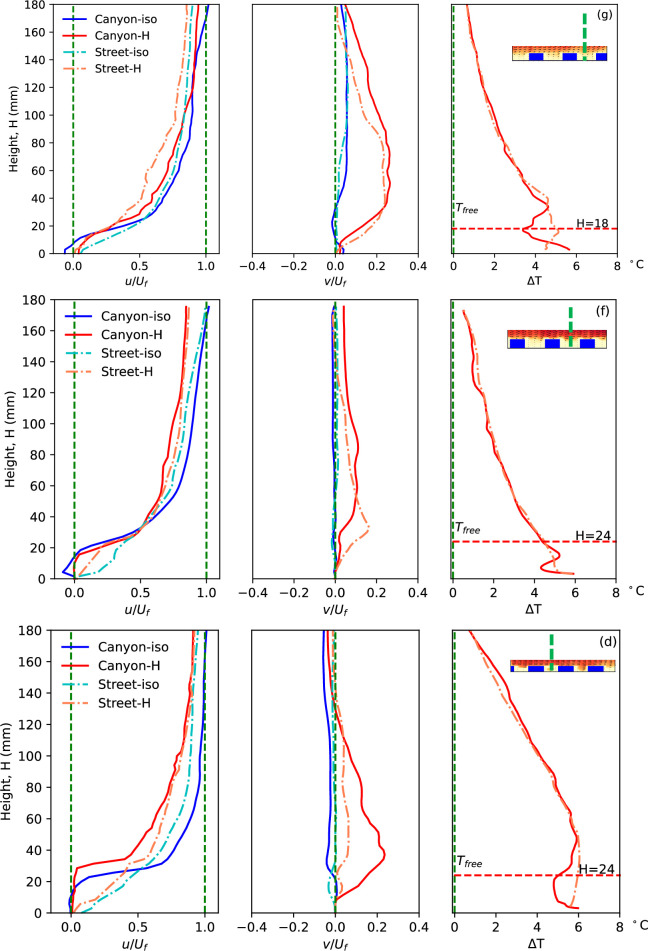
Normalized streamwise velocity, vertical velocity and the temperature rise along the canyon centreline in different urban morphologies case (g), (f) and (d). The blue dashed lines indicate the location of the plotted properties.

Temperature of flow in the urban area, both in the canyon and the street, is significantly influenced by canyon configuration, while temperature profiles over the canyon show good agreement between street and canyon regions. This temperature difference in the urban region is generated by different flow patterns in the canyon, such as the vortex structure. Similarly, with taller buildings and weaker influence from the main flow, more significant differences are observed, evidenced by good similarity of temperature rise in case (a) and significant difference in the lower region of case (b). In other words, the farther from the source, whether main freestream flow or heated ground surface, the less influence via flow pattern. It is also worth mentioning that there’s good agreement of flow property distribution along the canyon centreline presented in figures 7 and 8 with previously published results [31–36], although different configurations lead to slight differences. One of the main contributions of this work is the improved quality of high-resolution data.

Figure 6 compares the updraft buoyant flow at the canyon roof level in case (a) with low buildings and case (b) with high buildings, with the locations of the building models indicated by dashed blocks, also representing the canyon locations. In the isothermal flow condition, negligible vertical flow is observed along the roof level of both scenarios. However, peaks indicating strong updraft buoyant flow under the heating condition are observed at the canyon openings. As mentioned earlier, the development of buoyant flow along the streamwise direction due to the accumulation of thermal force is clearly captured in case (a), evidenced by the growing peaks of updraft buoyant flow along the canyon and street planes. However, this accumulation of thermal force is more significant in case (b) due to the higher building models, resulting in weaker influence from the heated ground surface. Despite the narrow street width, the flow over the building models can influence the flow over the street, resulting in a similar pattern of upwards flow along the street. By contrast, significant differences are observed in case (b), characterized by a wider street and higher building models. Here, the flow along the street does not exhibit corresponding updrafts, differing from the flow along the canyon plane. This discrepancy underscores the influence of urban morphology, particularly building height and street width, on flow dynamics and buoyant flow patterns.

**Figure 9.**
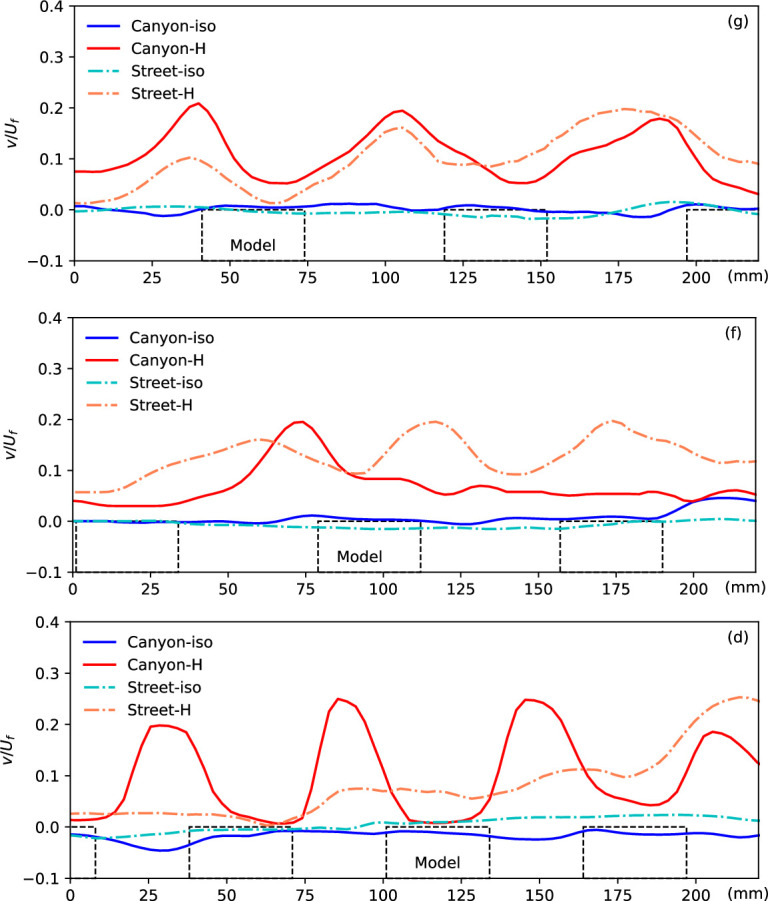
The normalized vertical velocity at the canyon roof level along the flow in different scenarios (g), (f) and (d), as indicated by the green dashed line in figure 5. The locations of the models are presented by the dashed blocks.

Cases (a) and (b) exhibit dramatically different heights of building models, but the widths of the canyon and street also vary. Hence, cases (g) (*H* = 18 mm, *W* = 45 mm) and (f) (*H* = 24 mm, *W* = 45 mm) provide useful insights into the diverse impacts of building height. In these cases, the building heights are 18 mm and 24 mm, respectively, while all other parameters, including a canyon width of 45 mm, remain constant. Figures 8 and 9 present the flow parameters along the canyon centrelines and roof level. It is noteworthy that the canyon centrelines of cases (g) and (f), indicated by the blue dashed lines in figure 8, represent the same locations as shown in figure 1, despite depicting different canyon locations. A notable similarity in streamwise flow is observed between the two cases. The decelerating flow in non-isothermal conditions and along the street in both cases aligns with the aforementioned cases.

In the isothermal condition, the presence of negative streamwise velocity in the lower region of the street canyon implies the existence of the canyon vortex. The influence of heated ground is evident in the significant updraft of buoyant flow, as indicated by the vertical velocity component. Case (g) exhibits a stronger updraft flow, attributable to its lower building height, resulting in a larger velocity and updraft region. This aligns with the earlier example of case (a), where a lower building model experiences a stronger influence of the thermal force, as it is closer to the thermal source and also has a larger local Richardson number [24]. Similarly, the flow pattern within the canyon contributes to the zigzag shape of temperature profiles along the canyon centreline in both cases. The stronger updraft of case (g) pushes more high-temperature fluid upwards, resulting in a more pronounced zigzag shape in temperature distribution.

A significant difference in vertical velocity along the roof level in the streamwise direction is evident in the tests with different model heights, as shown in figure 9. Comparing the updraft in case (g) to that in case (f), a distinct pattern emerges. In the non-isothermal flow of the higher model (f), only one strong updraft is observed at the first canyon, with no significant updraft from the other canyon openings. This variance in vertical flow performance along the roof level is attributed to the model height, specifically the canyon aspect ratio (*H*/*W*). As discussed in previous studies [12,31,37], in isothermal flow within street canyons, the canyon aspect ratio dominates the flow pattern, leading to isolated roughness flow, wake interference flow and skimming flow. In essence, whether the approaching flow enters and interacts with the flow in the canyon is determined by the canyon configuration. This observation is further supported by the current comparison with isothermal flow: case (g), with a canyon aspect ratio of 0.4, shows the entry of freestream flow into the canyon, as indicated by the negative vertical velocity at the canyon openings, whereas this phenomenon is not observed in case (f) with an aspect ratio of 0.53. This disparity in aspect ratio also influences the updraft performance in non-isothermal conditions. Along the street, where the flow pattern is strongly influenced by the flow at the canyon edges, these updrafts remain visible at roughly the corresponding canyon locations.

#### (ii) Flow parameters within urban areas with different canyon width

Results from case (f) (*H* = 24 mm, *W* = 45 mm) and (d) (*H* = 24 mm, *W* = 30 mm) shed light on the impacts of canyon width, where buildings of the same height are positioned at different densities, resulting in aspect ratios of 0.53 and 0.8, respectively. Despite having the same model height, these scenarios exhibit varying flow properties along the height, particularly in and near the canyon, as depicted in figure 8. The stronger updraft buoyant flow from the narrow canyon (case (d)) is evidenced by the significant positive vertical velocity (*v/U_f_*), which slightly elevates the streamwise mainstream flow, thickening the boundary layer over the canyon roof, as indicated by the streamwise velocity. This stronger updraft also expands the region of the flow impacted by the heated ground surface, resulting in higher temperatures and a thicker zigzag-shaped temperature distribution within the canyon. The vertical velocity of non-isothermal urban flow along the canyon centreline in the streamwise direction of case (d), shown in the bottom plot of figure 9, exhibits a similar distribution to case (g), with strong updrafts at each canyon opening, but differs from case (f). The vertical velocity along the street also differs from cases (g) and (f), showing no response to the canyon location but indicating the accumulation of thermal impacts, similar to case (a).

In isothermal flow, the aspect ratio of a street canyon can lead to various vortex structures within the canyon, such as two horizontal vortices, merging vortex, single vortex, or multiple vortices in the vertical direction, with an increase in aspect ratio [31,37]. Similarly, in non-isothermal flow, the canyon configuration, specifically the aspect ratio, dominates the flow pattern, influenced by the interaction between shear flow and buoyant flow. With the same flow conditions and shear stress, the total shear force acting on the canyon flow through the canyon opening is directly proportional to the cross-sectional area or width of the canyon in the streamwise direction. Consequently, there is a direct relationship between thermal effects and canyon configuration, evident from the temperature profiles. Thermal effects become more pronounced if the canyon has a large aspect ratio, common in high-density cities with narrow canyons and relatively high buildings. The buoyant flow in a low and wide canyon with a small aspect ratio of 0.4 and uniform height is strong enough to overcome the suppression of approaching flow, resulting in significant updrafts and enhanced ventilation and heat removal performance. However, determining the relationship between aspect ratio and flow pattern within the canyon and street, particularly considering canyon location, is complex. For instance, with an increase in canyon aspect ratio from 0.4 to 0.53 and 0.8 (cases (g), (f) and (d)), no clear tendency is observed, particularly with significant buoyancy-driven updrafts. A systematic study of aspect ratio focusing on its impact on flow pattern in non-isothermal urban flow is highly recommended.

### (c) Symmetry and asymmetry of the urban areas

**Figure 10.**
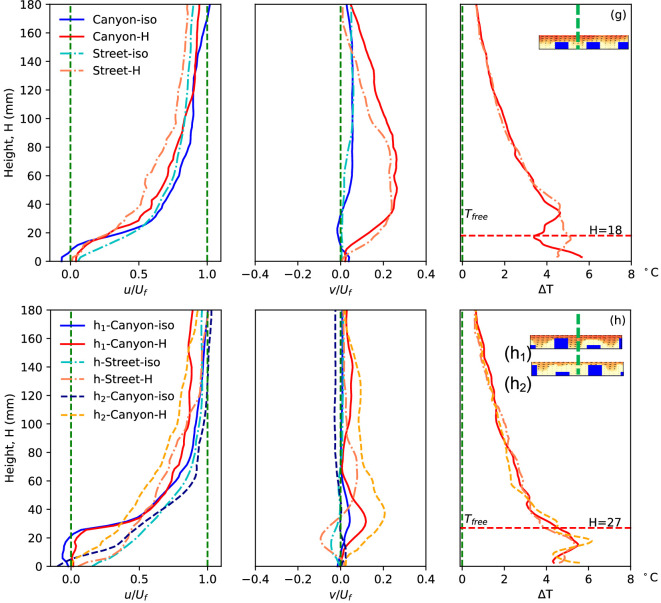
Normalized streamwise velocity, vertical velocity and the temperature rise along the canyon centreline in different urban morphologies case (g) and (h). The blue dashed lines indicate the location of the plotted properties.

In real urban scenarios, buildings typically vary in height rather than being uniform across an area. To illustrate the impact of this variation, we compare the flow properties at the canyon centre plane in cases (g) and (h), as shown in figure 10. Both cases have an aspect ratio of 0.4, with a canyon width of 45 mm. Case (g) consists of building models with a uniform height of 18 mm, while case (h) employs blocks with heights of 8 mm and 27 mm, resulting in an average height of 18 mm. The inset in figure 10 depicts the setup, with (*h*_1_) and (*h*_2_) representing the step-down and step-up canyons, respectively, in case (h).

Comparing the flow patterns in case (g) with those in case (h), we observe similar streamwise flow along the street plane but differences along the canyon planes. The varied streamwise centreline velocities in the canyon region explain the distinct flow patterns in the step-down (*h*_1_) and step-up (*h*_2_) canyons, as documented in [24]. Under non-isothermal conditions, vertical velocity indicates upward flow along the canyon centreline due to buoyant force. Conversely, flow along the street is drawn into the canyon, resulting in negative vertical velocity below the roof level. Due to these varying flow patterns, temperature profiles along the canyon centrelines in cases (g) and (h) show similar distributions within the canyon, while temperatures along the street are comparable.

In non-isothermal conditions, the canyon flow becomes buoyant due to convective heating from the ground surface, which inhibits the formation of the canyon vortex and results in significant updraft flow, especially when combined with the upward flushing flow. This intensified updraft contributes to higher vertical velocity along the canyon’s centreline. Additionally, the vector fields in the inset figures provide insights into the vertical velocity along the canyon roof level, revealing significant updrafts in the (*h*_2_-Canyon-H) configuration.

**Figure 11.**
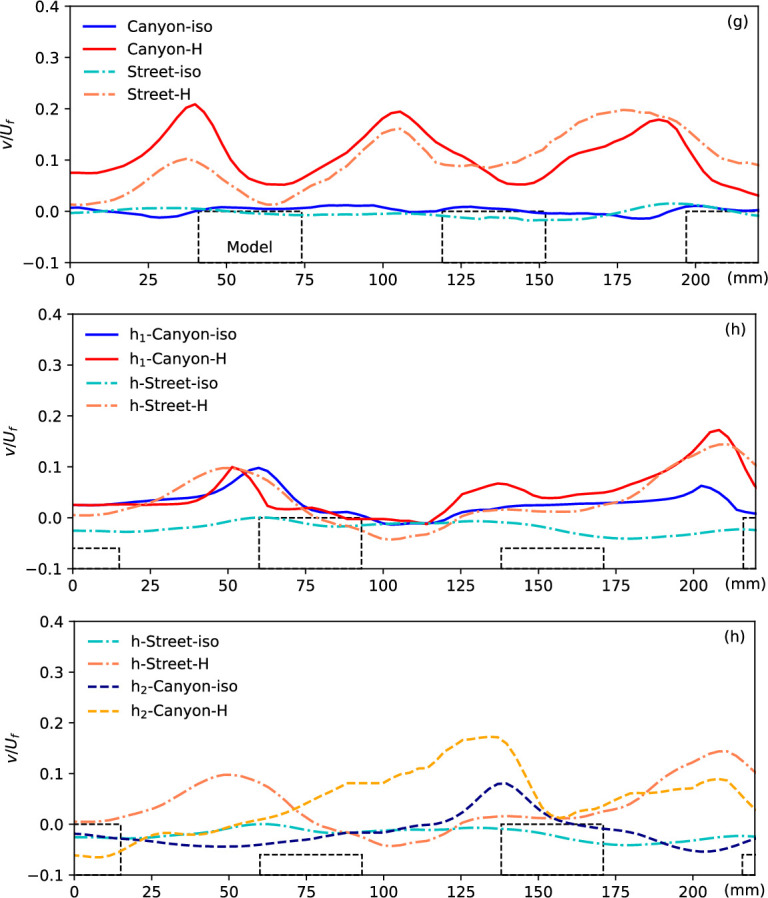
Normalized vertical velocity at the canyon roof level along the flow in different scenarios (g) and (h). The locations of the models are presented by the blocks (h_1_ and h_2_) along the two planes of the canyon centrelines as shown in figure 1(*h)*.

We delve deeper into the vertical velocity profiles along the canyon roof level, specifically at approximately 18 mm for case (g) and 27 mm for case (h), as depicted in figure 11. Here, the step-down canyon (h_1_) and step-up canyon (h_2_) are delineated by black dashed in the inset figures, respectively, offering relative location information for the vertical velocity profiles. Comparative analysis reveals notable distinctions between the strong non-isothermal updrafts observed at each canyon opening in case (g) and the impact of only the higher building models on flow in case (h), with minimal changes near the lower models. For instance, at *x* ≈ 50 and 220 mm in the (h_1_) plane and *x* ≈ 135 mm in the (h_2_) plane—positions corresponding to the edges of the higher blocks—significant buoyant updrafts, denoted by peak values of the vertical velocity, are evident. Conversely, negligible changes in vertical velocity near the lower models are observed.

The inclusion of lower building models in case (h) transforms the designated canyon into a wider configuration, its width now comprising the sum of the two original canyon widths and the building width, owing to the diminished impact of the lower buildings on flow. Furthermore, the presence of downward flow within the canyon region in the heated ground surface scenario suggests the existence of a complex horizontal flow pattern in the vicinity of the canyon.

Remarkably, in the isothermal condition, significant variation in vertical flow is observed across the urban area with non-uniform building heights, including downward flow along the street and canyon. This variance, distinct from the uniform building height scenario of case (g), is primarily attributed to changes in the aspect ratio of the canyon, where higher building blocks act as isolated rough elements. Flow along the street is heavily influenced by the two neighbouring canyons, with negative velocity along the street plane when the floor is heated, indicating buoyant flow downward to the floor and into the canyon from the side opening.

## 4. Ventilation rate of the canyons

The vertical component of the flow plays an important role in understanding the urban climate mitigation, as it tells the ventilation and heat removal performance. The volumetric ventilation rate of a cavity, *Q'* is calculated based on the instantaneous vertical velocity at the opening (*v*), expressed as:


(4.1) $$Q^{\prime }=\frac{\tau }{V_{c}}\int_{A}^{}vdA$$

where *τ* denotes the reference time and is calculated as $\tau =2(H+S)/(2U_{f}/3)$, *V_C_* represents the unit volume and *A* is the ventilation area of interest.

**Figure 12.**
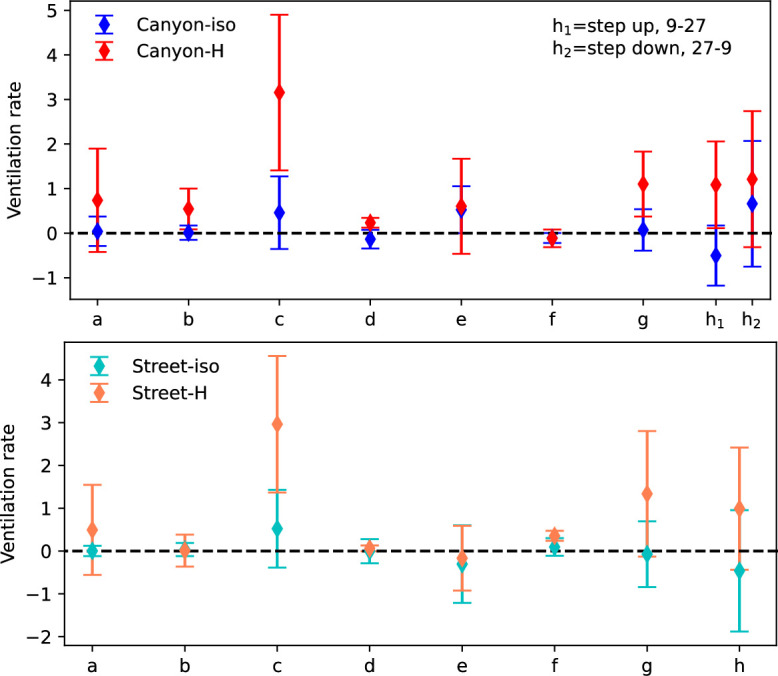
The ventilation rates from the canyon openings and same position of the street in different tested scenarios.

The temporospatial ventilation rates at the canyon roof level have been plotted to illustrate air ventilation patterns in both spatial and temporal directions, as reported in [18,28]. These results also highlight unsteady flow behaviour and the potential presence of periodic flow structures, such as plumes, evidenced by the fluctuating patterns. By averaging the ventilation rates across spatial and temporal directions, the calculated rates for all tested scenarios are summarized in figure 12, with error bars representing their variations. Significant differences are observed in both the ventilation rates and their fluctuations, which can vary by up to approximately 10.5 times and 12.2 times, respectively, under heating conditions. Noticeable increases in ventilation rate due to surface heating are observed in all cases except case (f), which exhibits almost negligible influence from the thermal factor. Similarly, the fluctuation of ventilation is also enhanced by the thermal source. Cases (b) (*H* = 84 mm, *W* = 18 mm), (d) (*H* = 24 mm, *W* = 30 mm) and (f) (*H* = 24 mm, *W* = 45 mm) demonstrate a steadier state, while the other cases all exhibit strong variation, with case (c) (*H*_1_ = 9 mm, *H*_2_ = 84 mm, *H*_3_ = 99 mm, *W* = 18 mm) displaying the most significant fluctuation. The step-up (h_1_) street canyon exhibits a faster ventilation rate in both isothermal and non-isothermal conditions than the step-down (h_2_). The relatively small ventilation rate in case (f) is attributed to the combined impacts of the canyon configuration and surface heating condition. The canyon flow in such a street canyon features a weak updraft component and suppression of the approaching flow.

The ventilation rates along the canyon roof level on the street are also computed, as shown in figure 12. Similarly, significant enhancement of ventilation is observed after the ground surface is heated (in cases (a) (*H* = 9 mm, *W* = 12 mm), (c), (g) (*H* = 18 mm, *W* = 45 mm), and (h) (*H*_1_ = 27 mm, *H*_2_ = 9 mm, *W* = 45 mm)). As the street flow is controlled by the buoyant flow and two neighbouring canyon flows, the canyon configuration, including canyon height, width, aspect ratio, and the variation in building height, can also impact the street flow. For cases with uniform building height ((b), (d) and (f)), ventilation rates are almost negligible regardless of the heating condition. Negative ventilation rates in case (e) (*H*_1_ = 36 mm, *H*_2_ = 12 mm, *W* = 30 mm) imply that flow is drawn from the canyon side opening into the canyon. Significant changes in ventilation in cases (c) and (h) (*H*_1_ = 27 mm, *H*_2_ = 9 mm, *W* = 45 mm), which have variable heights, demonstrate the impact of heating. Generally, the non-uniform height of the building models leads to more unsteady flow, particularly along the vertical direction in both the canyon and the street. Besides the canyon aspect ratio, the absolute height of the canyon, or more specifically the distance from the disturbing source to the mainstream, can also significantly impact the flow pattern and hence ventilation performance in non-isothermal conditions.

## 5. Conclusion and recommendation

This study offers a comprehensive exploration of non-isothermal urban flow dynamics across diverse urban morphologies, employing advanced PIV/LIF techniques within a controlled water tunnel environment. Our investigation aimed to elucidate the intricate interplay between urban morphology, thermal effects, and flow characteristics, with implications for urban climate and ventilation performance. By capturing the coupled effects of buoyancy-driven updrafts and ground heating, our findings provide new insights into how urban heat spreads both above the ground, reinforcing the importance of integrated urban climate studies.

Key insights gleaned from our experiments underscore the pivotal role of canyon aspect ratio in shaping flow patterns and ventilation efficacy within urban environments. Specifically, the aspect ratio emerges as a critical determinant, influencing the balance between shear flow and buoyant flow within street canyons. Our findings reveal that low and wide canyons with small aspect ratios exhibit robust buoyant updrafts, significantly enhancing ventilation rates. However, the relationship between building height and buoyant flow is nuanced, with taller buildings shown to mitigate the influence of buoyancy in certain scenarios. Interestingly, no consistent trend emerges across all cases, emphasizing the complexity of the interactions between urban morphology and thermal effects.

Furthermore, our investigations highlight the impact of variable building heights on flow patterns within street canyons. In particular, the formation of canyon vortices and subsequent flow dynamics are influenced by the distribution of building heights and the convective heating of the ground surface. While convective heating enhances buoyant flow, it also suppresses vortex formation, resulting in distinctive flow patterns under non-isothermal conditions. These findings contribute directly to the theme of urban heat spreading across scales by illustrating how different urban morphologies affect local thermal transport and ventilation performance. The influence of neighbouring canyons on street flow is also evident, underscoring the significance of urban symmetry in shaping urban microclimates. Buoyant updrafts observed at canyon openings correspond to coherent flow patterns along the street, reflecting the complex interactions between canyon morphology and thermal factors.

Despite the valuable insights provided by our experiments, it is important to acknowledge the inherent limitations. While laboratory experiments offer controlled conditions, they may not fully capture the complexities of real-world urban environments as discussed in [24]. Therefore, we advocate for complementary high-resolution CFD simulations to provide a more comprehensive understanding of urban morphology and thermal effects.

In conclusion, our study contributes to a deeper understanding of the multifaceted relationship between urban morphology, thermal effects and flow dynamics in non-isothermal urban environments. By elucidating these interactions, we provide valuable insights for informing sustainable urban design practices and climate mitigation strategies. Future research endeavours should prioritize systematic investigations to further elucidate the intricate interplay between urban morphology and thermal effects, thereby advancing our understanding of urban microclimate dynamics.

## Figures and Tables

**Figure 13.**
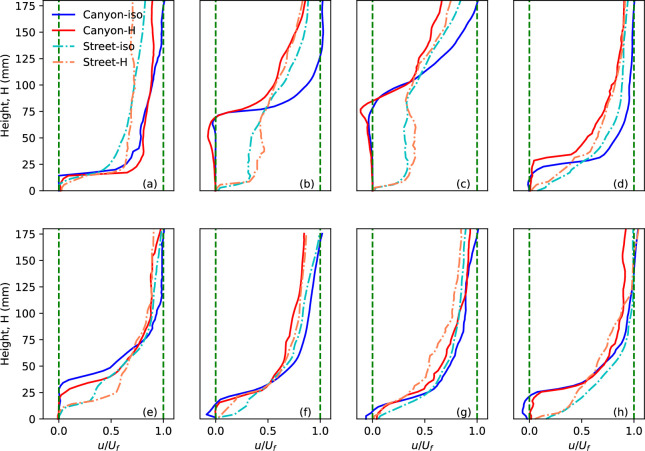
The normalized streamwise velocity along the centerlines in different scenarios.

**Figure 14.**
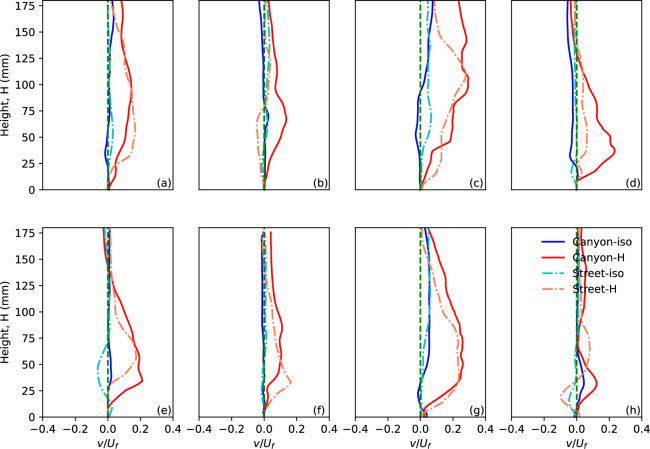
The normalized vertical velocity along the centrelines in different scenarios.

**Figure 15.**
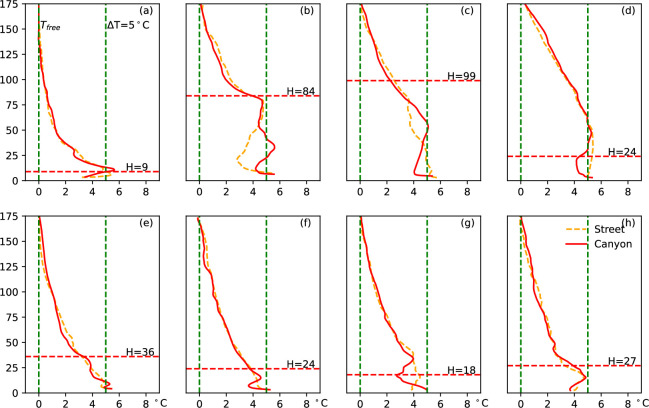
The temperature rise along the centrelines in different scenarios.

## Data Availability

The datasets generated during and/or analysed during the current study are available from the corresponding author on reasonable request.

## Appendix A.

To offer comprehensive insights into the flow patterns and properties observed in the various tested scenarios, streamwise velocity, vertical velocity and temperature profiles are provided in this appendix for reference (figures 13–15).

## References

[1] Manoli G, Fatichi S, Schläpfer M, Yu K, Crowther TW, Meili N, Burlando P, Katul GG, Bou-Zeid E. 2019 Magnitude of urban heat islands largely explained by climate and population. Nature **573**, 55–60. (10.1038/s41586-019-1512-9) 31485056

[2] Zhao L, Lee X, Smith RB, Oleson K. 2014 Strong contributions of local background climate to urban heat islands. Nature **511**, 216–219. (10.1038/nature13462) 25008529

[3] Perera ATD, Javanroodi K, Nik VM. 2021 Climate resilient interconnected infrastructure: co-optimization of energy systems and urban morphology. Appl. Energy **285**, 116430. (10.1016/j.apenergy.2020.116430)

[4] Santos LGR, Nevat I, Pignatta G, Norford LK. 2021 Climate-informed decision-making for urban design: assessing the impact of urban morphology on urban heat island. Urban Climate **36**, 100776. (10.1016/j.uclim.2021.100776)

[5] Zhao Y, Li R, Feng L, Wu Y, Niu J, Gao N. 2022 Boundary layer wind tunnel tests of outdoor airflow field around urban buildings: a review of methods and status. Renew. Sustain. Energy Rev. **167**, 112717. (10.1016/j.rser.2022.112717)

[6] Li S, Xiao Q, Teng M, Qiu X, Xu W, Liu H, Wu X, Wu C. 2023 A comprehensive morphological classification scheme for local ventilation performance zones in spatially heterogeneous urban areas. Dev. Built Environ. **15**, 100202. (10.1016/j.dibe.2023.100202)

[7] Yang C, Zhu W, Sun J, Xu X, Wang R, Lu Y, Zhang S, Zhou W. 2021 Assessing the effects of 2D/3D urban morphology on the 3D urban thermal environment by using multi-source remote sensing data and UAV measurements: a case study of the snow-climate city of Changchun, China. J. Clean. Prod. **321**, 128956. (10.1016/j.jclepro.2021.128956)

[8] Tian Y, Zhou W, Qian Y, Zheng Z, Yan J. 2019 The effect of urban 2D and 3D morphology on air temperature in residential neighborhoods. Landsc. Ecol. **34**, 1161–1178. (10.1007/s10980-019-00834-7)

[9] Du H, Perret L, Savory E. 2024 Effect of urban morphology and an upstream tall building on the scale interaction between the overlying boundary layer and a street canyon. Bound. Layer Meteorol. **190**, 5. (10.1007/s10546-023-00844-8)

[10] Lim HD, Hertwig D, Grylls T, Gough H, Reeuwijk M van, Grimmond S, Vanderwel C. 2022 Pollutant dispersion by tall buildings: laboratory experiments and large-eddy simulation. Exp. Fluids **63**, 92. (10.1007/s00348-022-03439-0) 35673586 PMC9165307

[11] Tolias IC, Koutsourakis N, Hertwig D, Efthimiou GC, Venetsanos AG, Bartzis JG. 2018 Large Eddy Simulation study on the structure of turbulent flow in a complex city. J. Wind Eng. Ind. Aerodyn. **177**, 101–116. (10.1016/j.jweia.2018.03.017)

[12] Coceal O, Thomas TG, Castro IP, Belcher SE. 2006 Mean flow and turbulence statistics over groups of urban-like cubical obstacles. Boundary Layer Meteorol. **121**, 491–519. (10.1007/s10546-006-9076-2)

[13] Javanroodi K, Nik VM, Giometto MG, Scartezzini JL. 2022 Combining computational fluid dynamics and neural networks to characterize microclimate extremes: learning the complex interactions between meso-climate and urban morphology. Sci. Total Environ. **829**, 154223. (10.1016/j.scitotenv.2022.154223) 35245539

[14] Zhang J, Li Z, Hu D. 2022 Effects of urban morphology on thermal comfort at the micro-scale. Sustain. Cities Soc. **86**, 104150. (10.1016/j.scs.2022.104150)

[15] Moon K, Hwang JM, Kim BG, Lee C, Choi J il. 2014 Large-eddy simulation of turbulent flow and dispersion over a complex urban street canyon. Environ. Fluid Mech. **14**, 1381–1403. (10.1007/s10652-013-9331-2)

[16] Aristodemou E, Boganegra LM, Mottet L, Pavlidis D, Constantinou A, Pain C, Robins A, ApSimon H. 2018 How tall buildings affect turbulent air flows and dispersion of pollution within a neighbourhood. Environ. Pollut. **233**, 782–796. (10.1016/j.envpol.2017.10.041) 29132119

[17] Chen G, Rong L, Zhang G. 2020 Comparison of urban airflow between solar-induced thermal wall and uniform wall temperature boundary conditions by coupling CitySim and CFD. Build. Environ. **172**, 106732. (10.1016/j.buildenv.2020.106732)

[18] Xue Y, Zhao Y, Mei SJ, Yuan C, Carmeliet J. 2024 The significant impacts of urban canyon configuration on non-isothermal flow, ventilation, and heat removal: insights from PIV-LIF measurements. Int. J. Heat Fluid Flow **110**, 109594. (10.1016/j.ijheatfluidflow.2024.109594)

[19] Wai KM, Yuan C, Lai A, Yu PKN. 2020 Relationship between pedestrian-level outdoor thermal comfort and building morphology in a high-density city. Sci. Total Environ. **708**, 134516. (10.1016/j.scitotenv.2019.134516) 31806333

[20] Allegrini J, Dorer V, Carmeliet J. 2015 Influence of morphologies on the microclimate in urban neighbourhoods. J. Wind Eng. Ind. Aerodyn. **144**, 108–117. (10.1016/j.jweia.2015.03.024)

[21] Allegrini J, Dorer V, Carmeliet J. 2015 Coupled CFD, radiation and building energy model for studying heat fluxes in an urban environment with generic building configurations. Sustain. Cities Soc. **19**, 385–394. (10.1016/j.scs.2015.07.009)

[22] Allegrini J, Carmeliet J. 2017 Coupled CFD and building energy simulations for studying the impacts of building height topology and buoyancy on local urban microclimates. Urban Clim. **21**, 278–305. (10.1016/j.uclim.2017.07.005)

[23] Allegrini J. 2018 A wind tunnel study on three-dimensional buoyant flows in street canyons with different roof shapes and building lengths. Build. Environ. **143**, 71–88. (10.1016/j.buildenv.2018.06.056)

[24] Xue Y, Zhao Y, Mei SJ, Chao Y, Carmeliet J. 2024 Impact of street canyon morphology on heat and fluid flow: an experimental water tunnel study using simultaneous PIV-LIF technique. Exp. Therm. Fluid Sci. **150**, 111066. (10.1016/j.expthermflusci.2023.111066)

[25] Zhao Y, Xue Y, Mei S, Chao Y, Carmeliet J. 2022 Enhancement of heat removal from street canyons due to buoyant approaching flow: water tunnel PIV-LIF measurements. Build. Environ. **226**, 109757. (10.1016/j.buildenv.2022.109757)

[26] Yuan C, Adelia AS, Mei S, He W, Li XX, Norford L. 2020 Mitigating intensity of urban heat island by better understanding on urban morphology and anthropogenic heat dispersion. Build. Environ. **176**, 106876. (10.1016/j.buildenv.2020.106876)

[27] Zhang L, Yuan C. 2023 Multi-scale climate-sensitive planning framework to mitigate urban heat island effect: a case study in Singapore. Urban Clim. **49**, 101451. (10.1016/j.uclim.2023.101451)

[28] Xue Y, Zhao Y, Mei SJ, Chao Y, Carmeliet J. 2024 Exploring thermal buoyant flow in urban street canyons: influence of approaching turbulent boundary layer. Exp. Therm. Fluid Sci. **158**, 111255. (10.1016/j.expthermflusci.2024.111255)

[29] Mori E, Quadrio M, Fukagata K. 2017 Turbulent drag reduction by uniform blowing over a two-dimensional roughness. Flow Turbul. Combust. **99**, 765–785. (10.1007/s10494-017-9858-2)

[30] Demarco G *et al*. 2022 Analysis of thermal and roughness effects on the turbulent characteristics of experimentally simulated boundary layers in a wind tunnel. Int. J. Environ. Res. Public Health **19**, 5134. (10.3390/ijerph19095134) 35564529 PMC9104942

[31] Lin Y, Ichinose T, Yamao Y, Mouri H. 2020 Wind velocity and temperature fields under different surface heating conditions in a street canyon in wind tunnel experiments. Build. Environ. **168**, 106500. (10.1016/j.buildenv.2019.106500)

[32] Jiang G, Yoshie R. 2018 Large-eddy simulation of flow and pollutant dispersion in a 3D urban street model located in an unstable boundary layer. Build. Environ. **142**, 47–57. (10.1016/j.buildenv.2018.06.015)

[33] Allegrini J, Dorer V, Carmeliet J. 2014 Buoyant flows in street canyons: validation of CFD simulations with wind tunnel measurements. Build. Environ. **72**, 63–74. (10.1016/j.buildenv.2013.10.021)

[34] Duan G, Ngan K. 2019 Sensitivity of turbulent flow around a 3-D building array to urban boundary-layer stability. J. Wind Eng. Ind. Aerodyn. **193**, 103958. (10.1016/j.jweia.2019.103958)

[35] Marucci D, Carpentieri M. 2020 Stable and convective boundary-layer flows in an urban array. J. Wind Eng. Ind. Aerodyn. **200**, 104140. (10.1016/j.jweia.2020.104140)

[36] Castro IP, Xie ZT, Fuka V, Robins AG, Carpentieri M, Hayden P, Hertwig D, Coceal O. 2017 Measurements and computations of flow in an urban street system. Bound. Layer Meteorol. **162**, 207–230. (10.1007/s10546-016-0200-7)

[37] Oke TR. 1988 Street design and urban canopy layer climate. Energy Build. **11**, 103–113. (10.1016/0378-7788(88)90026-6)

